# Medical and Physiological Commentaries

**Published:** 1840-12

**Authors:** 


MEDICAL AND PHYSIOLOGICAL COMMENTARIES. BY MARTYN PAYNE, M D., A. M.

We have received a copy of this work, which is one of the largest original publications on medicine that the American press has yet brought forth. An early review of it will be prepared by one of our ablest collaborators. Y.

